# Anti-microbial, anti-oxidant, and anti-breast cancer properties unraveled in yeast carotenoids produced *via* cost-effective fermentation technique utilizing waste hydrolysate

**DOI:** 10.3389/fmicb.2022.1088477

**Published:** 2023-01-18

**Authors:** Sweta Sinha, Souvik Das, Biswajit Saha, Debarati Paul, Biswarup Basu

**Affiliations:** ^1^Amity Institute of Biotechnology, Amity University, Noida, India; ^2^Department of Neuroendocrinology and Experimental Hematology, Chittaranjan National Cancer Institute, Kolkata, West Bengal, India

**Keywords:** yeast carotenoids, mandi waste, sustainable production, breast cancer therapeutics, *in vitro* and *in silico* study

## Abstract

**Introduction:**

Natural carotenoids are well known for their anti-oxidant property and also shown to have antimicrobial and anticancer efficacy. Production of carotenoids from microbial resources mainly from yeast has attracted commercial interest. Breast cancer has the highest incidence among women, and therapy resistance and lack of effective therapeutic strategies are major treatment bottlenecks, particularly for triple-negative subtypes. Yeast carotenoids are recently being evaluated for affordable, non-toxic, natural product-based therapies. In the present study, we have shown an environment-friendly and inexpensive method for carotenoid production from yeasts, utilizing “mandi” wastes, and investigated the biomedical properties of carotenoids, particularly antineoplastic properties.

**Methods:**

Vegetable “mandi” waste was used to prepare waste hydrolysate, a culture medium, in which oleaginous red yeast *Rhodosporidium* sp. was grown. Carotenoid pigments were extracted using the solvent extraction method and analyzed by UV spectroscopy, thin-layer chromatography (TLC), and high-performance liquid chromatography (HPLC). Antimicrobial, antioxidant, and anticancer activities of the extract were evaluated, followed by in silico docking and absorption, distribution, metabolism, and excretion/toxicity (ADME/T) studies.

**Results:**

Carotenoid extract was found to be composed of three main pigments-β-carotene, torulene, and torularhodin. Extract exhibited significant antioxidant, antimicrobial, and anti-breast cancer activities *in vitro* while being biocompatible. Interestingly, carotenoids have shown better efficacy in triple-negative breast cancer (TNBC) cells than ER+PR+ cells. *In silico* evaluation predicted binding with breast cancer-specific molecular targets, specifically the three components showed good binding energy toward VEGF receptors and good drug likeliness properties, as well as less toxicity.

**Discussion:**

This is the first report on anti-breast cancer activities, particularly targeting TNBC cells by red yeast carotenoids (β-carotene, torulene, and torularhodin) produced via a sustainable environment-friendly bioprocess utilizing waste hydrolysate.

## Introduction

The antioxidant in carotenoids helps to inhibit the initiation of carcinogenesis, modify bio-membranes, reinforce the lipid bilayer, and decrease its fluidity (Black et al., 2020). Natural carotenoids (e.g., β-carotene) help body defense by scavenging reactive oxygen species (ROS), in the human body. Carotenoids also up-regulate gap junction communication, induce detoxifying enzymes, and inhibit cell proliferation (Shin et al., 2020). Biomedical application of carotenoids includes antimicrobial activity as shown by lutein, astaxanthin, etc. (Neumann et al., 2019). Increased intake of carotenoids and elevated blood levels of β-carotene have shown reduced risks of lung cancer (Niranjana et al., 2015). Earlier reports emphasized the anti-breast cancer activity of carotenoids by inhibiting proliferation and inducing apoptosis in breast cancer cell lines such as MCF7 and MDAMB 231 through up-regulation of ROS, DNA fragmentation, caspase 3 activation, Bcl 2 down-regulation, and by modulating other important cellular pathways (Black et al., 2020; Koklesova et al., 2020; Saini et al., 2020), but breast cancer-specific receptor targeting is not well known.

Chemically synthesized carotenoids are carcinogenic; thereby, their production from microbial resources is endorsed. Yeasts utilize cheap feedstock (e.g., carbonaceous waste), which results in lowering the production cost plus yielding useful by-products. Among microbes, yeast carotenoid biosynthesis has attracted commercial interest compared to algae or fungi, as yeasts are unicellular and exhibit a high growth rate to facilitate large-scale fermentation (Mata-Gómez et al., 2014). Among the yeast strains, the species belonging to *Phaffia*, *Rhodosporidium*, etc., genera produce key carotenoid pigments, e.g., torulene, β-carotene, and torularhodin (Mata-Gómez et al., 2014; Chreptowicz et al., 2019). Plant-based carotenoid consumption showed lowered cancer risks and enhanced the immune system (Mignone et al., 2009), while antioxidant and antibacterial efficacy were reported in yeast carotenoids extracted from *Rhodotorula glutinis* (Fiedor and Burda, 2014).

Many commercial breast cancer drugs currently developed resistance in patients, and there are no effective therapies for some aggressive breast cancers like triple negative subtypes. Hence, there is a dire need for affordable, non-toxic, natural product based anti-breast cancer drug development, and therefore, yeast carotenoids may be excellent choices.

The benefits of yeast carotenoids include low-cost production, non-dependency on arable land, fertilizers, and climate and hence should be explored for potential biomedical application. This study is the first evaluation of the anti-breast cancer property of yeast carotenoids grown using a fermentation medium derived from waste hydrolysates, the bioprocess of which may be considered environmentally friendly and inexpensive.

## Material and methods

### Preparation of waste hydrolysate

Discarded and uneatable parts of vegetables and fruits were collected from various roadside markets (mandis), in Delhi/NCR region, India. These included cauliflower/cabbage stubs, peels of peas, the skin of banana/oranges/sweet lime, and fruit parts. The waste was frozen after collection and segregation, to prevent the loss of sugar. The carbon (C) and nitrogen (N) content of the waste material was determined individually so that they could be mixed in appropriate proportions to achieve the desired C/N ratio of 20:1, which has been standardized by our earlier study (Sinha et al., 2020). After weighing the waste (30 g), the peels were chopped, added to water (100 ml), and steamed under pressure in an autoclave, to prepare about 100 ml of the hydrolysate as reported in detail earlier (Singh et al., 2018).

### Cultivation of yeast on waste hydrolysate

The organism used in this study was *R. toruloides* ATCC 204091 (Sinha et al., 2020). Peels of various seasonal fruits and inedible parts of vegetables (stubs of cabbage and cauliflower, pea pods) were collected from the local vegetable markets (“mandis”), to prepare a hydrolysate Waste Extract (WE) and used as fermentation medium. Optimal nitrogen C:N ratio of 20:1 was maintained in the WE medium by mixing appropriate amounts of high carbon containing fruit waste. After filtering and cooling to room temperature, the hydrolysate was used as a culture medium. Fermentation of yeast was carried out in the 500-ml shake flasks, each containing 100 ml cultivation media, and was inoculated with a starter culture (10% v/v) and incubated at 30°C for 120 h, as described earlier (Sinha et al., 2020).

### Extraction, quantitation, and analysis of carotenoids

**FIGURE 1:**
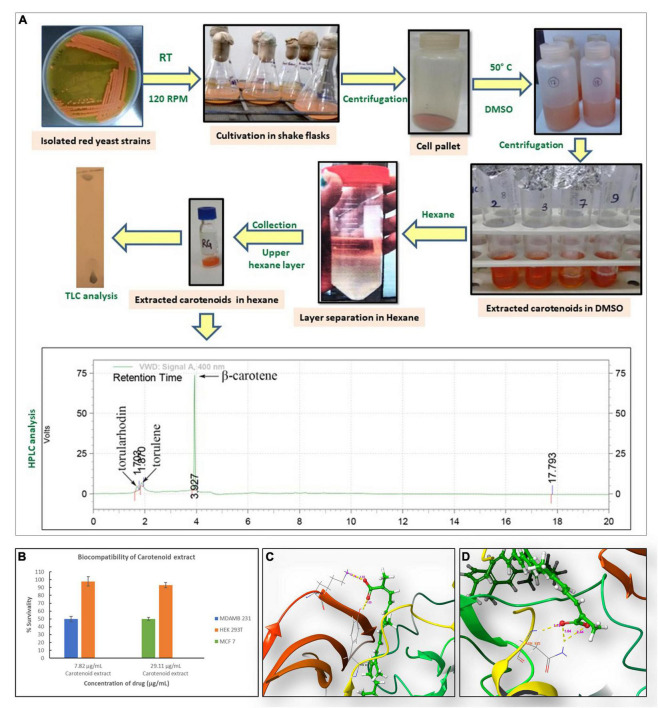
**(A)** Flow diagram for extraction of yeast carotenoids and its analysis. **(B)** Survivability analysis of carotenoid extract in HEK 293T with respect to IC_50_ concentrations of MCF7 (29.11 μg/ml) and MDA-MB-231 (7.82 μg/ml). HEK293T cells showed insignificant changes in proliferation. Results are representative of three independent experiments. **(C)** 3D interaction diagram between torularhodin and vascular endothelial growth factor receptor 1 (VEGFR1) showing hydrogen bond formed with asparagine 923 at the active site of the protein and carboxyl group moiety of torularhodin. **(D)** 3D interaction diagram between torularhodin and VEGFR2 showing hydrogen bond formed with lysine 831 and tyrosine 911 at the active site of protein and carboxyl group moiety of torularhodin.

The carotenoid was extracted from yeast cells by solvent extraction following the protocol given by Hiscox and Israelstam (1980), after suitable modifications (Figure 1A; Sinha et al., 2020). Optical density (OD) was determined *via* Systronics UV-Vis spectrophotometer at 400 nm against hexane. Quantitative analysis was done by means of a standard curve of β-carotene (Sinha et al., 2020). Thin-layer chromatography (TLC) analysis was carried out using silica gel 60 TLC plates (Merck, Germany) and petroleum ether and acetone (80:20 v/v) as a mobile phase and Rf values were determined as described earlier (Marova et al., 2010). The carotenoids were also separated and analyzed by high-performance liquid chromatography (HPLC) on Agilent, C_18_ column, and a gradient solvent system of water: acetonitrile was used for separation of pigments, followed by detection at 250 nm (UV-Visible Detector).

### Antimicrobial activity

The antimicrobial property was evaluated by the agar diffusion method where wells on a Nutrient Agar plate were loaded with 20 μL of carotenoid extract (10 mg/ml). The plate was previously spread with 10^8^ CFU/ml of microbial suspension. After incubation at 37°C for 24 h, inhibition zones around the spot were measured in millimeters. Wells contained only hexane (negative control) and 20 μL of 1.0 mg/ml antibiotic ciprofloxacin (positive control). Both gram-positive and gram-negative bacteria were used as mentioned in the table (Supplementary Table 1) for testing antibacterial activity. Two yeast strains, i.e., *Candida albicans* (MTCC 183), and *Saccharomyces cerevisiae* (MTCC 170), and fungal strains, i.e., *Fusarium oxysporum* (MTCC 2773) and *Aspergillus niger* (MTCC 281) were used to test antimicrobial activity. For positive control in the case of yeast and fungal strains, amphotericin B was used (20 μg/ml). The % inhibition was calculated by taking the diameter of the antibiotic inhibition zone as 100%.

### Antioxidant activity

2,2-Diphenyl-1-picrylhydrazyl (DPPH) radical scavenging activity was determined by taking various concentrations of the carotenoid extract (10–50 μg/ml), exposing it to 1.5 ml freshly prepared solution of DPPH in methanol for 20 min, and then measuring the absorbance at 515 nm. The percentage inhibition of DPPH in the medium was calculated by comparing it with the control (untreated DPPH). Percentage inhibition was calculated as below:


$$I\,n\,h\,i\,b\,i\,t\,i\,o\,n\%=\frac{\left(O\,D_{D\,P\,P\,H}-O\,D_{S\,a\,m\,p\,l\,e}\right)}{O\,D_{D\,P\,P\,H}}\times 100$$

### Cell culture and *in vitro* cellular cytotoxicity assay

Cell culture reagents were procured from Gibco (Thermo Fischer Scientific, Inc., USA). Two human breast cancer cell lines, MCF7 and MDA-MB-231, and one human normal cell line HEK 293T were procured from NCCS, Pune, India. Breast cancer cells were cultured, and cellular cytotoxicity was evaluated against carotenoid extracts treated in increasing concentrations, as described earlier (Selvendran et al., 2021). Graphical plotting of concentration vs. OD values was analyzed, and inhibition of proliferation by the extract was compared to vehicle-treated control. IC_50_ values (concentration showing 50% proliferation inhibition) for carotenoid extract were calculated for both MCF-7 and MDA-MB-231 cells, and the extract was further evaluated in normal cells (HEK 293T) with corresponding IC_50_ concentrations to check whether the anti-proliferative potency is restricted to cancer cell line only (de Souza et al., 2021; Fatima et al., 2021; Kaur et al., 2021).

### *In silico* molecular docking study

*In silico* molecular docking studies were performed to understand possible receptor mediated anti-breast cancer pathways through which the active molecules in the carotenoid extract may exert anti-proliferative activity. Torularhodin, torulene, and β-carotene structures were docked into the active site of several human breast cancer receptors *via* estrogen receptor (ERα, ERβ), progesterone receptor (PR), human epidermal growth factor receptor 2 (HER2/ErBB2), vascular endothelial growth factor receptor 1 and 2 (VEGFR1 and VEGFR2), fibroblast growth factor receptors (FGFR1 and FGFR2), and EGFR. Protein structures were downloaded from Research Collaboratory for Structural Bioinformatics (RCSB)^1^ protein data bank, followed by a protein preparation wizard where pre-process (assigning bond order, adding missing hydrogen, filling missing loop, and missing side chain) and refinements (optimization of H bond, minimization using OPLS4 force field) were done. Ligand structures were downloaded from the PubChem database and prepared using the LigPrep wizard (charge fixation, orientation of groups, 2D to 3D conversion, corrected bond lengths and bond angles, adding hydrogens) using the OPLS4 force field. Receptor grid and task grids were generated on proteins and ligands were docked using the ligand docking task of the GLIDE application (Maestro 12.8, Schrödinger Suite- 2021-3).

### *In silico* absorption, distribution, metabolism, excretion (ADME), and toxicity studies

To evaluate drug likeness and pharmacological profile, the QikProp application was used, which reports a 95% range for known drugs with several descriptors and properties of Schrödinger workflow to evaluate *in silico* ADME properties. The probability of toxicity was also predicted using admetSAR online tool (Cheng et al., 2012).

### Statistical analyses

All assays were performed in triplicates and expressed as mean ± standard deviation (SD). Data analysis was performed with the software package Microsoft Excel, version 2016. Statistically significant difference was determined using one-way Analysis of Variance (ANOVA). and *p* ≤ 0.05 was used as a limit to indicate statistical significance.

## Results

### Growth of red yeast strain, extraction, and analysis of carotenoid

To grow the red yeast strain, a medium was prepared using “mandi” waste consisting of inedible peels of fruits and unused parts of vegetables. Fruit and vegetable peels having a high content of carbon were added to waste containing appropriate nitrogen content, to maintain the C/N ratio to 20:1 in the WE that promotes carotenoid accumulation over lipid production (Sinha et al., 2020). The maximum amount of carotenoid (62 ± 0.93 mg/L) was produced by *R. toruloides* at 88 h of growth in the above medium. The extracted pigments were separated using acetone:petroleum ether (20:80) as mobile phase (Figure 1A). TLC results showed that the Rf value of a band from the extract (0.90) was similar to that of a standard β-carotene spot (Yehia et al., 2013). Three main pigments (i) β-carotene, (ii) torulene, and (iii) torularhodin were visually separated as also shown earlier (Park et al., 2007; Minyuk and Solovchenko, 2018). HPLC analysis confirmed the above finding where β-carotene appeared at 3.9 min and torulene and torularhodin at 1.8 and 1.7 min (Figure 1A), whereas other smaller peaks remained unidentified. Several studies on carotenoid production from yeast strains on different mediums have been reported previously, showing that the waste of different types, e.g., cassava-bagasse, sago-starch hydrolysate, and whey, (Sinha et al., 2020) is suitable for cultivation of oleaginous red yeast for carotenoid production. Here, we developed a culture medium using discarded vegetable parts and grown oleaginous red yeast *Rhodosporidium* sp. Carotenoid extract analysis revealed mainly three major compounds, i.e., β-carotene, torulene, and torularhodin, similar to HPLC analysis observed for *R. glutinis* grown under external stress (Marova et al., 2010).

### The carotenoid extract showed antimicrobial and antioxidant activities

The pink lipid soluble carotenoid extract was found to be very effective against both gram-negative and gram-positive strains and yeast. It was, however, not effective against fungal cultures. Comparing diameters of the inhibition zone (Supplementary Table 1) showed that it was most active against *Escherichia coli* and *Pseudomonas* sp. as compared to *Staphylococcus aureus*, *Micrococcus*, *Candida* sp., etc., The carotenoid extract was able to scavenge stable free radical DPPH and its IC_50_ was 25 ± 5 μg/ml (Supplementary Figure 1). Earlier reports, using DPPH assay, showed comparable IC_50_ values for astaxanthin 79.32 ± 18.10, lutein 35, and zeaxanthin 10 μg/ml (Neumann et al., 2019).

### The carotenoid extract showed selective cytotoxicity in breast cancer cells

We were further compelled to evaluate the anti-proliferative properties of the extract as many antioxidant natural compounds have shown anticancer activities and are being pursued as anticancer drug candidates (Kaur et al., 2021). When screened against human breast cancer cell lines, *in vitro*, the carotenoid extract showed dose-dependent inhibition of proliferation and IC_50_ in MCF7 and MDA-MB-231 cells were calculated as 29.11 and 7.82 μg/ml, respectively (Supplementary Figure 2). The carotenoid extract was also found to be biocompatible as we obtained insignificant cytotoxicity in normal human cells HEK293T when treated with these IC_50_ concentrations separately (Figure 1B). These results indicated the selective anti-proliferative potency of carotenoid extract in breast cancer cells.

### The carotenoid extract showed breast cancer-specific target binding *in silico*

The major chemotherapeutic drugs used nowadays to suppress breast cancer cells are developed for targeted delivery of chemo-agents against several molecular and cellular signaling pathway proteins, mainly, for example, ER, PR, HER2, VEGFR1, VEGFR2, and EGFR (Sarkar and Mandal, 2009; Jin and Ye, 2013). We conducted *in silico* molecular docking to evaluate whether the active compounds of the extracts, i.e., β-carotene, torulene, and torularhodin can bind or interact with the breast cancer-specific major molecular targets. After their molecular docking with several breast cancer-specific cellular targets, binding energy was calculated (ΔG, reported as kcal/mol) and listed (Table 1). Among all, molecular interaction between VEGFR2 and torularhodin reflects the highest binding energy (−10.092 kcal/mol) followed by VEGFR1 > ERα > ERβ > HER2. Torularhodin formed a hydrogen bond with asparagine 923 at the active site of the protein VEGFR2 and the carboxyl group moiety of the molecule. A similar type of interaction was also found between torularhodin and VEGFR1 and between lysine 831 and tyrosine 911 of the VEGFR1 active site (Figures 1C,D). The binding energy of torulene was highest with VEGFR2 (−9.217 kcal/mol) followed by VEGFR1 > ERα. The binding energy for β carotene in decreasing order was found as HER2 > VEGFR2 > VEGFR1 > ERα. Our results indicated that the selective anti-proliferative activity may be governed through binding with cellular tyrosine kinase receptor VEGFR (VEGFR1 and VEGFR2). Interestingly expansion and regulation of the breast cancer stem cell population directly depend on VEGFR2 expression (Zhao et al., 2014; Farzaneh Behelgardi et al., 2020), and inhibitors against VEGFR1 and VEGFR2 have been designed to suppress tumor growth and under the clinical application. According to a recent study, it has been demonstrated in animal breast cancer models, that carotenoids as phytochemicals showed anti-proliferative properties through VEGFR down-regulation (Metibemu et al., 2021).

**TABLE 1:** Calculated binding energy of torularhodin, torulene, and β-carotene.

Breast cancer cellular targets (PDB ID)	Binding energy (Δ G in kcal/mol)		
	**Torularhodin**	**β -carotene**	**Torulene**
ERα (1SJ0)	−8.746	−8.256	−8.100
ERβ (1NDE)	−8.277	−7.269	−7.967
PR (1E3K)	No pose	No pose	No pose
HER2/ErBB2 (3PP0)	−8.250	−8.906	−7.910
VEGFR1 (3HNG)	−9.932	−8.474	−8.304
VEGFR2 (3VHE)	−10.092	−8.728	−9.217
FGFR1 (6C18)	−7.945	−6.555	−6.433
FGFR2 (7OZY)	−6.220	−5.856	−5.605
EGFR (3QWQ)	−5.286	−4.351	−4.467
**Pharmacological properties**	**Calculated values**		
Molecular weight	564.85	534.867	536.882
DonorHB	1	0	0
AcceptHB	2	0	0
Rule of five	2	2	2
Rule of three	2	2	2
QPPCaco (nm/s)	324.457	9,906.038	9,906.038
QPlogBB	−2.259	2.229	2.199
HOA (%)	100	100	100
CNS	−2	2	2
**Toxicity properties**			
AMES mutagenesis	−	−	−
Mitochondrial toxicity	+	−	−

In silico Pharmacological properties and toxicity of torularhodin, torulene, and β-carotene. To determine drug-likeness properties, parameters like molecular weight (mol_WT), number of hydrogen bonds that would be donated by the solute to water molecules in an aqueous solution (donorHB), and would be accepted by the solute from water molecules in an aqueous solution (acceptHB) were calculated in silico. For QPlogBB, the suitable range is −3.0–1.2. MDCK cell permeability (QPPMDCK) and Caco2 cell permeability (QPPCaco), are mimics of blood brain barrier and gut blood barrier model, respectively (value < 25 considered as poor and > 500 nm/s is great). Active compounds for the central nervous system (CNS, −2 considered inactive, whereas+2 as active) were evaluated. The probability of human oral absorption percentage (HOA %, >80% is considered as high) was also calculated for three compounds. AMES toxicity (the ability of a chemical or drug to induce mutations in DNA) and mitochondrial toxicity were also evaluated.

### Pharmacological properties and toxicities of carotenoid extract components

Several properties were evaluated *in silico* for drug likeness and toxicities of torularhodin, torulene, and β-carotene (Table 1). All showed good molecular weight (∼500 kDa), with donor and accept HB values within suitable range (below 5) and not violating the rule of five (maximum being 4) and rule of three (maximum being 3). Among the three compounds, the predicted brain/blood partition coefficient (QPlogBB) of torularhodin was good. The probability of crossing the blood–brain barrier and gut–blood barrier was high for torulene and β carotene while torularhodin was moderate. Torulene and β-carotene were shown to be active compounds in central nervous system (CNS). The probability of human oral absorption percentage (HOA %) was high for all, besides all being non-toxic (AMES toxicity negativity probability > 75%).

## Discussion

Fruit/vegetable waste from mandis and cafeterias/shops are mostly carried to composting sites. They are not sorted and are simply dumped in the composting site. The management and disposal patterns may include landfilling and soil improvement additives. They may also be converted to bioactive or further converted *via* biotechnological routes to high value-added chemicals and materials (Alexandri et al., 2020). In our study, red yeast *Rhodosporidium* sp. was cultured in a medium prepared from discarded parts of vegetables. From this oleaginous yeast, carotenoids were extracted using the solvent extraction method. Further HPLC analysis revealed that the carotenoid extract is mainly represented by three major compounds, i.e., β-carotene, torulene, and torularhodin. The extract has shown good antimicrobial and antioxidant properties on preliminary bioactivity analysis. It is also evident that the products of all fungal enzymes and metabolites are beneficial for human health as they are involved in food and pharma products (Shankar and Sharma, 2022). Their importance has been mainly applied due to their immunomodulatory (Fukushima-Sakuno, 2020), anticancer (Ball et al., 2020), anti-inflammatory (Xu et al., 2018), and antiviral properties (Wisbeck et al., 2017). According to our *in vitro* evaluation of the anticancer property of carotenoid extract against breast cancer cells, significant anti-proliferative properties were observed, but no significant proliferation inhibition was evident in normal cells, indicating selective efficacy in breast cancer cells. Interestingly, here, we report better anti-proliferative efficacy in basal type MDA-MB-231 cells (triple negative, ER^–^, PR^–^, HER2^–^) compared to luminal type MCF7 cells (ER^+^, PR^+^, HER2^–^). Carotenoids have already been shown to have anti-cancer effects including breast cancer properties (Prakash et al., 2001; Gloria et al., 2014). In relation to our findings, from a recent publication, it was demonstrated that the β-carotene, one of the major components of the carotenoid extract, found to be anti-proliferative and stimulate apoptosis and also cause cell cycle arrest in triple-negative breast cancer (TNBC) cell line MDA MB 231, MDA MB 235, and MCF7 *in vitro* (Antunes et al., 2022). It is also evident that when β-carotene is treated in combination with doxorubicin, the dose of inhibition concentration against breast cancer cell line got reduced remarkably (Vijay et al., 2018). In another previous study, through ameliorating the anti-oxidant mechanism, β-carotene inhibited the growth of the MCF7 cell line (Sowmya Shree et al., 2017). Other two major components of carotenoid extract, i.e., torulene and torularhodin, have also been demonstrated to have anti-prostate cancer activity through induction of apoptosis as reported from a series of *in vitro* and *in vivo* studies (Du et al., 2016, 2017).

To understand the mechanism of proliferation inhibition, we did virtual binding of major constituents of extracts (β-carotene, torulene, and torularhodin) with major molecular targets of breast cancer and also evaluated drug likeliness properties *in silico*.

As per our *in silico* docking study, the binding energy for all three components from the extracts is significantly higher for VEGFR. From the binding energy, it can be considered that among all three components, specifically, torularhodin can be an important molecule for targeting TNBC subtypes, targeting through the VEGFR. There is a need for novel effective therapies for the treatment of patients with TNBC due to its aggressive, chemotherapy resistant nature, and extremely poor prognosis; hence, several drugs have been developed against overexpressed EGFR, VEGFR, and FGFR for TNBC targeting which are already in clinical practice or shown promising results in clinical trials (Tafreshi et al., 2020). It has also been established *in vivo* that carotenoid, through VEGFR down-regulation, exhibits anti-proliferative properties in breast carcinoma (Metibemu et al., 2021).

Our *in silico* findings also indicated that carotenoid extract has good pharmacological properties to be considered as a drug candidate, and their selective anti-proliferative properties may be governed through binding with cellular tyrosine kinase receptor VEGFR (VEGFR1 and VEGFR2). Yeast carotenoid could be a potentially promising therapeutic approach, as indicated by our initial finding that can be investigated further.

## Conclusion

The cost-effective cultivation of oleaginous red yeast using hydrolysate of fruit/vegetable waste for producing carotenoids may be considered a zero-waste process and gives a good return on investment. The bioprocess is designed such that there is minimal waste production because both the biomass and the mineral rich fermentation media may be used for enhancing agriculture in nearby farms. The antioxidant and anti-cancer properties of the natural yeast pigment have been established in this study using *in silico* and *in vitro* assays. The yeast carotenoid extract showed good efficacy on aggressive breast cancer cell lines and may be suggested as a replacement for synthetic carotenoids or plant-based/algal pigments, which are expensive, compete with agricultural land, are difficult to extract, and show lesser yield comparatively.

## Data availability statement

The original contributions presented in this study are included in this article/Supplementary material, further inquiries can be directed to the corresponding authors.

## Author contributions

SS and SD performed the experiments. BS performed the analytical studies. BB and DP conceptualized the study, analyzed the data, and were responsible for writing and proofreading the manuscript. All authors contributed to the article and approved the submitted version.

## Footnotes

1
https://www.rcsb.org/
