# Erratum: Anti-microbial, anti-oxidant, and anti-breast cancer properties unraveled in yeast carotenoids produced *via* cost-effective fermentation technique utilizing waste hydrolysate

**DOI:** 10.3389/fmicb.2023.1193497

**Published:** 2023-04-04

**Authors:** Frontiers Production Office

**Affiliations:** Frontiers Media SA, Lausanne, Switzerland

**Keywords:** yeast carotenoids, mandi waste, sustainable production, breast cancer therapeutics, *in vitro* and *in silico* study

Due to a production error, the text “One of the handling editors for this special issue is DP, who is also an author of this submitted manuscript. We have therefore suggested name of other handling editor Shashi Kant Bhatia for our manuscript to avoid the conflict.” was incorrectly added to the Conflict of Interest Statement. The corrected statement reads:

“The authors declare that the research was conducted in the absence of any commercial or financial relationships that could be construed as a potential conflict of interest.”

The publisher apologizes for this mistake. The original article has been updated.

