# Supplementary material for: Anti-microbial, anti-oxidant, and anti-breast cancer properties unraveled in yeast carotenoids produced via cost-effective fermentation technique utilizing waste hydrolysate
Source: Front Microbiol. 2023 Jan 18;13:1088477. doi: 10.3389/fmicb.2022.1088477 (PMC9889640; doi:10.3389/fmicb.2022.1088477)
Supplement: Supplementary file 1 [file Table_1.pdf]

Supplementary tables and Figures:

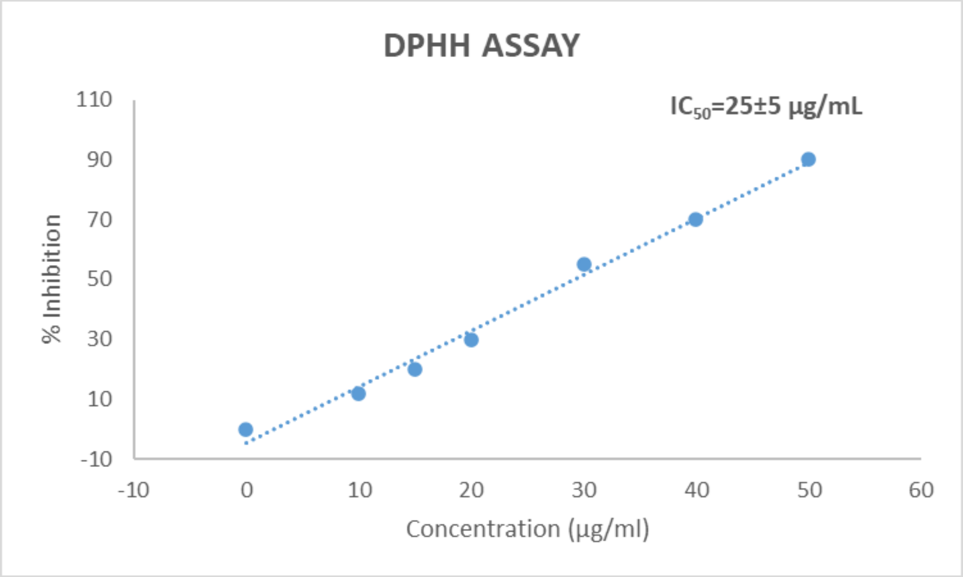

Supplementary Figure 1. Scatter plot analysis of DPPH assay showing the IC_{50} value of carotenoid extract.

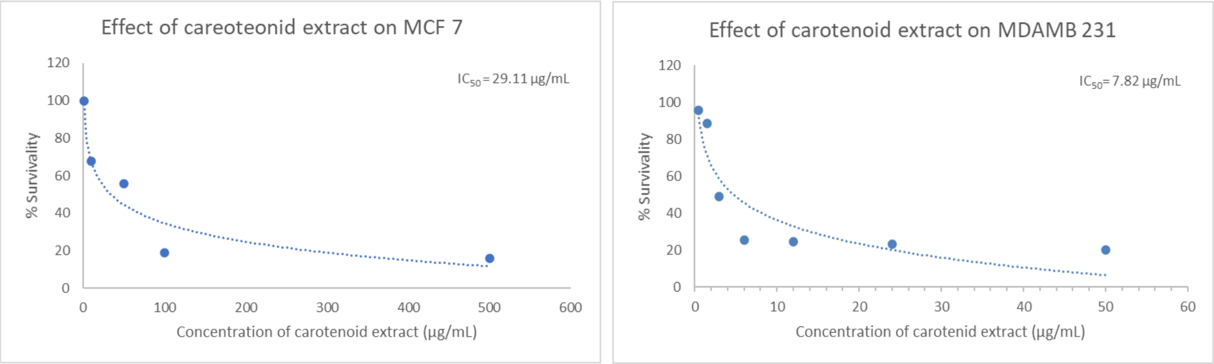

Supplementary Figure 2. Representative result of MTT assay showing significant dose-dependent inhibition of proliferation by carotenoid extract in breast cancer cell line MCF7 and MDA-MB-231. Experiments were repeated twice, IC_{50} values were calculated, and after statistical analysis, were found to be 29.35±0.82 µg/ml (IC_{50} for MCF7) and 7.85±0.21 µg/ml (IC_{50} for MDA-MB-231).

Supplementary Table 1.

**Strain**	**Inhibition zone diameter (mm)**	**% Inhibition**
Staphylococcus aureus (MTCC 1430)	12.5±0.2	69.4
Bacillus firmus (MTCC 488)	14.3±0.2	79.4
Bacillus subtilis (MTCC 121)	13.3±0.3	74.07
***Escherichia coli* (MTCC 1610)**	17.6±0.3	90.5
B. licheniformis (MTCC 429)	13.4±0.2	83.75
Listeria monocytogenes (MTCC 839)	10.2±0.3	59.8
B. thuringiensis (MTCC 1953)	12.5±0.5	69.4
Micrococcus luteus (MTCC 106)	10±0.3	55.4
***Pseudomonas aeruginosa* (MTCC 1934)**	15.6±0.4	82.1
Candida albicans (MTCC 183)	7±0.1	41.5
Saccharomyces cerevisiae (MTCC 170)	7.1±0.2	41.9
Fusarium oxysporum (MTCC 2773)	--	--
Asperigillus niger (MTCC 281)	--	--

Supplementary Table 1. Showing antimicrobial property of the extracted lipidic carotenoids. One way ANOVA results showed that the difference between the means were significant as $p \leq 0.05$.
